# Physiological and molecular profiling unveils oat (*Avena sativa* L.) defense mechanisms against powdery mildew

**DOI:** 10.3389/fpls.2025.1580472

**Published:** 2025-05-08

**Authors:** Aijie Ma, Tao Liu, Wenhui Tian, Hong Chen, Guoqing Wang, Bo Zhang

**Affiliations:** ^1^ Key Laboratory of Adaptation and Evolution of Plateau Biota, Northwest Institute of Plateau Biology, Chinese Academy of Sciences, Xining, Qinghai, China; ^2^ Qinghai Province Key Laboratory of Crop Molecular Breeding, Xining, Qinghai, China; ^3^ University of Chinese Academy of Sciences, Beijing, China; ^4^ Academician Workstation of Agricultural High-Tech Industrial Area of the Yellow River Delta, National Center of Technology Innovation for Comprehensive Utilization of Saline-Alkali Land, Dongying, Shandong, China

**Keywords:** oat, powdery mildew, disease resistance, ROS, SA

## Abstract

Oat powdery mildew, caused by *Blumeria graminis* f. sp. *avenae* (*Bga*), poses a serious threat to oat production, yet the underlying resistance mechanisms remain largely unclear. In this study, we investigated early-stage defense responses in resistant (BY642) and susceptible (BY119) oat varieties following *Bga* inoculation using cytological observations, physiological and hormonal measurements, and transcriptomic analysis. Microscopy revealed that Bga penetrates oat tissues directly through epidermal cells rather than stomata. BY642 exhibited a rapid and robust defense characterized by reactive oxygen species (ROS) accumulation and hypersensitive response, tightly regulated by an efficient antioxidant system to prevent cellular damage. Hormone profiling indicated a salicylic acid (SA)-dominated signaling pathway in BY642, accompanied by suppression of jasmonic acid (JA) responses. Transcriptome profiling showed early activation of photosynthesis-related pathways, metabolic reprogramming, and immune-related networks, including MAPK cascades and WRKY transcription factors. Hub genes such as *AsGSTU6* and *AsWRKY50* were identified as key contributors to resistance. These findings suggest that BY642 employs a coordinated defense strategy integrating ROS dynamics, SA signaling, and transcriptional regulation, providing novel insights into the molecular basis of powdery mildew resistance and potential targets for oat breeding.

## 1 Introduction

Cultivated oat (*Avena sativa* L.) is a globally cultivated crop, mainly distributed across Asia, Europe, and North America. Oats are rich in protein, lipids, dietary fiber and antioxidants, which can promote human growth and development, enhance the immune system and prevent diseases (Kim et al., 2021; Paudel et al., 2021). The high content of *β*-glucan in caryopsis can help reduce the risk of diet-related hypertension (Pins et al., 2002) and type 2 diabetes (Tapola et al., 2005). In addition, oat is a kind of excellent forage (Trevaskis et al., 2022) and can also be used to improve degraded and saline lands (Liu et al., 2020), which has many ecological benefits.

Oat powdery mildew caused by the biotrophic fungus *Blumeria graminis* f. sp. *avenae* (*Bga*) is one of the most common and destructive diseases of oat (Takamatsu, 2004). A significant reduction in cereal yields can be achieved by reducing the number of fertile tillering and the weight of grains (Clifford, 1995). Global agricultural losses attributed to powdery mildew have exceeded 8 million hectares annually since the onset of the 2020s, marking a significant threat to crop productivity worldwide (Liu et al., 2024). In the United States, yield losses caused by powdery mildew have been reported to range from 20% to 33% (Golzar et al., 2024). As a typical airborne disease, the spread of oat powdery mildew is usually affected by humidity and temperature; it has higher incidence and wider spread due to global warming (Chowdhury et al., 2014). Developing disease-resistant varieties is a cost-effective and environment-friendly approach to reduce the occurrence of oat powdery mildew. To date, only 13 powdery mildew resistance genes (*Pm1~Pm13*) have been found in oats (Mohler, 2022; Ociepa and Okoń, 2022; Schurack et al., 2024). And with the mutation of the pathogen, fewer genes found in the past can be used in practical oat production. Thus, it is of great significance to explore new genes for powdery mildew resistance in oats and to elucidate their molecular and physiological mechanisms.

In the long-term evolutionary process, close interactions between plants and pathogens have been formed. The plant’s primary physical defenses against biotic stress comprise specialized structures such as trichomes, multi-layered epidermal protections, along with wound-responsive callose formation (Singh et al., 2021). Pathogens invade plants through epidermal cells (Irieda and Takano, 2021) or natural openings such as stomata (Yang et al., 2021). The plant epidermis, functioning as the primary defensive interface, orchestrates disease resistance through multi-layered structural synergies. The cuticular waxes and cutin matrix form the fundamental physical barrier, impeding pathogen adhesion via hydrophobicity and mechanical resilience. Further reinforced by epidermal heterogeneity—including cell wall thickening, lignin deposition, stomatal density modulation with dynamic aperture regulation, morphological specialization of lenticels/hydathodes, and spatial distribution of sclerenchyma cells—this system establishes a selective filtration network to restrict pathogen penetration (Wan et al., 2021). As a dynamic defensive hub, the plant cell wall integrates mechanical support and signal transduction through its cellulose microfibril scaffold, hemicellulose cross-linked networks, pectin matrix, and structural proteins (Zhang et al., 2021). Beyond facilitating intercellular communication, it serves as a critical platform for pathogen recognition and immune activation. Upon detection of cell wall damage-associated molecular patterns (DAMPs) generated by pathogen-secreted cell wall-degrading enzymes (CWDEs) such as cellulases and pectate lyases, plants initiate a coordinated defense cascade.

Upon recognizing the invasion of pathogens, plants promptly activate their immune system. Research showed plants have evolved two immune systems to defend against infection. The first layer is PTI (pattern-triggered immunity), defined as plant immunity triggered by pathogen-associated molecular patterns (PAMPs), which are detected and recognized by pattern recognition receptors (PRRs) on the cell surface (Bigeard et al., 2015). However, pathogens have evolved to evade or suppress PTI through secreted effector molecules, which result in effector-triggered susceptibility (ETS) (Saur et al., 2019). To cope with this, plants’ intracellular nucleotide-binding leucine-rich repeat receptors (NLRs) would detect effectors, which are often encoded by Resistance (*R*) genes, and activate the second layer effector-triggered immunity (ETI). The interaction between PTI, ETS, and ETI has been incorporated into the widely cited “zig-zag-zig” intellectual framework (Ngou et al., 2022). Both PTI and ETI are accompanied by a set of induced defenses that repel pathogen attacks (Cui et al., 2015), including transient calcium signaling, production of ROS (reactive oxygen species) such as singlet oxygen (^1^O_2_), superoxide anion (O_2_^·−^), hydrogen peroxide (H_2_O_2_), and hydroxyl radical (HO^·^), playing a multifaceted role in plant defense mechanisms against pathogens (Patykowski and Urbanek, 2003; Waszczak et al., 2018), phosphorylation of Mitogen-activated protein kinase (MAPK) pathway (Meng and Zhang, 2013), hypersensitive response (HR) and activation of defense-related genes (Li et al., 2021), transcription factors (TFs), including WRKY, MYB, NAC, bZIP, and ERF, play pivotal roles as master regulators in controlling the expression of defense-related genes (Viswanath et al., 2023). Studies showed that key components in PTI and ETI are mutually required, and their synergistic interaction enhances immune responses (Chang et al., 2022).

The functions of plant hormones in response to viral infection have been widely studied, recent research showed that plant hormones play in defense regulation as a whole (Zhao and Li, 2021). Salicylic acid (SA) (Ding and Ding, 2020), jasmonic acid (JA) (Gupta et al., 2020), abscisic acid (ABA) (Kumar and Dasgupta, 2024), IAA (indole acetic acid) (Kunkel and Johnson, 2021) and gibberellic acid (GA) (Kumar et al., 2021) play key roles in modulating plant-virus interactions. These hormones signaling pathways are not simple linear or isolated cascades but intertwined and complex.

Although numerous studies have explored disease resistance mechanisms in plants, the specific resistance mechanism to powdery mildew in oat remains largely unknown. In this study, we identified BY642 as highly resistant to powdery mildew across all growth stages, while BY119 was clearly susceptible. To better understand the basis of this contrasting response, we investigated the early-stage physiological and molecular responses of both accessions following *Bga* inoculation. Through transcriptomic and functional enrichment analyses, we identified key signaling pathways and candidate genes potentially involved in regulating resistance to powdery mildew in oat.

## 2 Materials and methods

### 2.1 Growth conditions and inoculation

Hexaploid oat line BY642 (Aberglen, PI584829) exhibited a high level of all-stage resistance to powdery mildew, and BY119 (Victory) were highly susceptible to powdery mildew by the field and greenhouse identifying. Seedlings were grown in a greenhouse under controlled conditions (day/night 25°C/22°C, light/dark cycle16 h/8 h, 650 μmol m^-2^·s^-1^ light intensity, ~65% RH). Powdery mildew infection was performed as previously reported (Consonni et al., 2006) with some modifications. Two-week-old seedlings were drop-inoculated with *Blumeria graminis* f. sp. *avenae* (*Bga*). First, a layer of water mist was sprayed on the leaves to enhance spore adhesion, followed by gently shaking diseased oat leaves over the experimental plants. Powdery mildew resistance was assessed according to a previously described method (Ociepa et al., 2020), with slight modifications. Disease symptoms were evaluated 14 days post-inoculation. Visual assessment revealed that BY642 exhibited complete immunity, while BY119 was highly susceptible.

### 2.2 Observation of pathogen morphology

Leaves harvested from 6 hpi (hours post inoculation), 12 hpi, 24 hpi, 48 hpi, 72 hpi and 7 dpi (days post inoculation) were soaked in 0.4% trypan blue stain solution, boiled for 2 min and then transferred to 95% ethanol, boiled for 10 minutes to remove chlorophyll, and then imaged using a Olympus Fluorescence Microscopes BX53 under 10x or 40x. Observation of pathogenic microorganisms *in vivo* was used by a portable microscopy (Anyty portable microscopy 3R-MSUSB601, Japan). Safranine O-Fast Green Staining was used to observe leaf sections, leaves at 7 dpi were fixed in FAA solution, dehydrated through an ethanol series, cleared in xylene, and embedded in paraffin (Li et al., 2023b). Sections were cut, mounted, and deparaffinized in xylene, followed by rehydration through descending ethanol gradients. Staining was performed using 0.5% Safranin O and 0.1% Fast Green, with differentiation in acid-alcohol and ethanol rinses to remove excess stain. Sections were dehydrated, cleared, and mounted with a resinous medium for microscopic examination.

### 2.3 Histochemical detection of ROS

The formation of hydrogen peroxide in leaves was investigated via DAB staining (Daudi and O’Brien, 2012). Leaves harvested from 0 hpi, 3 hpi, 6 hpi, 12 hpi, 24 hpi, 48 hpi and 72 hpi were soaked in 1 mg/mL DAB, vacuum dried for 30 min and incubated overnight in the dark. The samples were then transferred to 95% ethanol, boiled for 10 minutes to remove chlorophyll, and then imaged under the Olympus Fluorescence Microscopes BX53 (40x). The detection of plant ROS (reactive oxygen species) levels was conducted via the fluorescence probe-based approach as previously outlined, employing H_2_DCFDA as the ROS-sensitive fluorescent probe (Fichman et al., 2019), plant optical imaging system NEWTON 7.0 Bio was used to detect and quantify ROS.

### 2.4 Determination of MDA and TAC

The same part of each leaf sample was harvested at 0 hpi, 3 hpi, 6 hpi, 12 hpi, 24 hpi, 48 hpi and 72 hpi and 7 dpi with three biological replicates, the samples were stored at -80°C for quantify MDA (malondialdehyde) (KTB9050, Abbkine) and TAC (total antioxidant capacity) (KTB1500, Abbkine) via instruction of manufacturers (Lin et al., 2024). All experiments were repeated at least three times.

### 2.5 Phytohormone quantification

Phytohormones were extracted using acetonitrile and purified with QuEChERS sorbents (C18 and GCB). The extracts were concentrated under nitrogen gas, re-dissolved in methanol, and filtered for analysis. Quantification of hormones, including IAA (indole acetic acid), ABA (abscisic acid), JA (jasmonic acid), SA (salicylic acid), and GA (gibberellic acid), was performed using HPLC-MS/MS on a PE QSight 420 triple quadrupole system in MRM mode (Almeida-Trapp and Mithöfer, 2019). Calibration curves were prepared with isotopically labeled internal standards to ensure accuracy and reproducibility. Leaves from 8 to 10 oat plants were pooled to measure hormone content at each time point.

### 2.6 RNA extraction, library construction and sequencing

The same part of each leaf sample was harvested at 0 hpi, 6 hpi, 12 hpi, 24 hpi, 48 hpi and 156 hpi with three biological replicates, the samples were stored at -80°C for RNA sequencing and Reverse transcription-quantitative polymerase chain reaction (RT-qPCR) analysis. Samples collected at six time points were processed to extract total RNA via a TRIzol reagent kit (Invitrogen, Carlsbad, CA, USA) (Guo et al., 2022). After purification and quality testing, the mRNA was converted to cDNA with reverse transcriptase. The final cDNA library was constructed and sequenced via paired-end sequencing technology with a read length of 150 bp on an Illumina NovaSeq 6000 instrument by Gene Denovo Biotechnology Co. (Guangzhou, China).

### 2.7 Data quality control and sequence comparative analysis

Reads obtained from the sequencing machines includes raw reads containing adapters or low quality bases which will affect the following assembly and analysis. Thus, to get high quality clean reads, reads were further filtered by fastp (version 0.18.0) (Chen et al., 2018). The parameters were as follows: (1) removing reads containing adapters; (2) removing reads containing more than 10% of unknown nucleotides (N); (3) removing low quality reads containing more than 50% of low quality (Q-value ≤ 20) bases. Short reads alignment tool Bowtie2 (version 2.2.8) (Langmead and Salzberg, 2012) was used for mapping reads to ribosome RNA (rRNA) database. The rRNA mapped reads then will be removed. The remaining clean reads were further used in assembly. An index of the reference genome was built, and paired-end clean reads were mapped to the reference genome using HISAT2.2.4 (Asativa_sang1.0) (Kim et al., 2015) with “-rna-strandness RF” and other parameters set as a default.

### 2.8 Differentially expressed genes and enrichment analysis

The mapped reads of each sample were assembled by using StringTie v1.3.1 (Pertea et al., 2015, 2016) in a reference-based approach. For each transcription region, a FPKM (fragment per kilobase of transcript per million mapped reads) value was calculated to quantify its expression abundance and variations, using RSEM (Li and Dewey, 2011) software. RNAs differential expression analysis was performed by DESeq2 (Love et al., 2014) software between two different groups. The genes/transcripts with the parameter of false discovery rate (FDR) below 0.05 and absolute fold change ≥2 were considered differentially expressed genes/transcripts. Gene Ontology (GO) (Ashburner et al., 2000) enrichment analysis provides all GO terms that significantly enriched in DEGs comparing to the genome background, and filter the DEGs that correspond to biological functions. Firstly, all DEGs were mapped to GO terms in the Gene Ontology database (http://www.geneontology.org/), gene numbers were calculated for every term, significantly enriched GO terms in DEGs comparing to the genome background were defined by hypergeometric test. The calculated p-value were gone through FDR Correction, taking FDR ≤ 0.05 as a threshold. GO terms meeting this condition were defined as significantly enriched GO terms in DEGs. Pathway-based analysis helps to further understand genes biological functions. Kyoto Encyclopedia of Genes and Genomes (KEGG) (Kanehisa and Goto, 2000) is the major public pathway-related database. Pathway enrichment analysis identified significantly enriched metabolic pathways or signal transduction pathways in DEGs comparing with the whole genome background. The calculated p-value was gone through FDR Correction, taking FDR ≤ 0.05 as a threshold. Pathways meeting this condition were defined as significantly enriched pathways in DEGs.

### 2.9 RT-qPCR analysis

cDNA was reverse transcribed via the PrimeScript FAST RT reagent Kit with gDNA Eraser, and RT-qPCR was performed via the TB Green^®^ Premix Ex Taq™ II FAST qPCR Kit. The *AsActin* gene was used as an internal control. The relative expression levels of genes were calculated via the 2^-ΔΔCT^ method. The primer sequences are listed in **Supplementary Table S1**.

### 2.10 Weighted gene co-expression network analysis

WGCNA (weighted gene co-expression network analysis) (Langfelder and Horvath, 2008) was performed in R to identify co-expressed gene modules. Low-expression genes were filtered out, and the soft threshold power (β) was determined using the scale-free topology criterion (R² > 0.80) and low mean connectivity. A correlation matrix was constructed and transformed into a topological overlap matrix (TOM). Genes were clustered based on TOM dissimilarity (1-TOM) using average hierarchical clustering and identified into modules with the dynamic tree cut algorithm (minimum cluster size of 30, merging threshold 0.25). Module membership (MM) was calculated as the Pearson correlation between genes and module eigengenes to identify hub genes. Network visualization was completed using Cytoscape (version 3.10.1).

### 2.11 Data processing and analysis

Statistical comparisons were performed using two-tailed Student’s t-tests. All datasets contained a minimum of three independent biological replicates, with each replicate derived from separate experimental batches. Calculations were executed in OriginPro 2025 using unpaired parametric implementation. Statistical significance was defined at *P* < 0.05, with exact P-values reported to two decimal places. R studio, OriginPro 2025 and Adobe Photoshop 2024 were used to generate graphs.

## 3 Results

### 3.1 Infection process of *Bga* in oats

**Figure 1:**
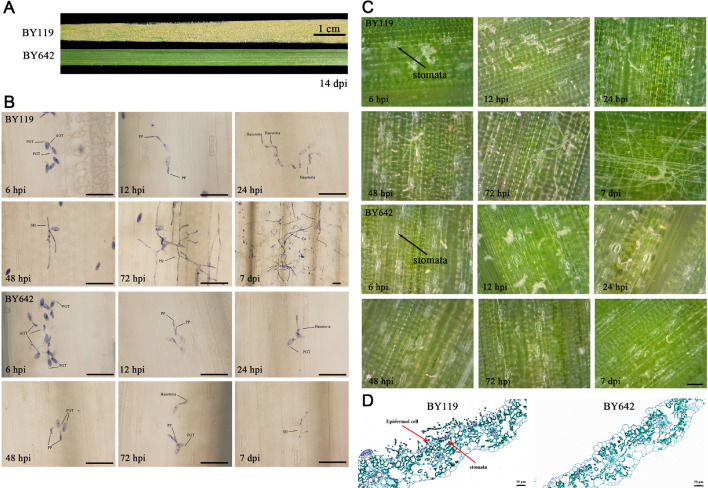
Infection process of powdery mildew on BY119 and BY642. **(A)** Phenotypic comparison of BY642 (resistant) and BY119 (susceptible) leaves at 14 dpi. **(B)** Trypan blue staining showing the infection stages of *Blumeria graminis* on BY119 and BY642 at different time points (10x or 40x). The dark bar indicates 50 μm. (PGT, primary germ tube; AGT, appressorium germ tube; PP, penetration peg; SH, secondary hyphae; Hy, hyphae; Co, conidia.) **(C)** Time-lapse observation of the infection process using a portable microscope. The dark bar indicates 50 μm. **(D)** Cross-sectional view of leaf tissue stained with safranin and fixed green, highlighting infection structures.

To understand the infection process and resistance response in two oat lines, we examined the development of *Blumeria graminis* f. sp. *avenae* (*Bga*) on BY119 (susceptible) and BY642 (resistant) following inoculation. It was found that powdery mildew spots appeared on the leaf surface of BY119 around 156 hours post inoculation (hpi), and at 14 days post inoculation (dpi), the leaves were completely covered with mycelium of powdery mildew. In contrast, no disease spots were observed on BY642 (**Figure 1A**). To monitor early fungal development, we conducted trypan blue staining to compare infection progression between the two lines. We found that the developmental progress of *Bga* was similar by 24 hpi (**Figure 1B**). At 6 hpi, both lines showed primary germ tubes (PGT) and appressorium germ tubes (AGT). By 12 hpi, penetration pegs (PP) were observed, and at 24 hpi, the penetration pegs elongated, indicating a tendency to invade epidermal cells, with haustoria also present. From 48 hpi onwards, mycelial growth was inhibited on BY642, while secondary mycelium (SM) developed on BY119. By 72 hpi, extensive mycelial growth was observed on BY119, further promoting fungal expansion. At 7 dpi, BY119 showed clear disease symptoms, with abundant mycelium and conidia covering the leaf surface. To investigate whether the pathogen entered through stomata, we observed stomatal behavior on living plants during infection. At all observed time points, the stomata remained closed, and no evidence of pathogen entry through the stomata was detected on the leaf surface (**Figure 1C**). Finally, to confirm the invasion route of *Bga*, we performed cross-sectional observation of infected leaves at 7 dpi. The results confirmed that *Bga* invaded oat tissues through epidermal cells rather than stomata on BY119 (**Figure 1D**). In summary, these findings indicate that while *Bga* can initiate early infection structures on both BY119 and BY642, resistance in BY642 halts further fungal development, and *Bga* invades susceptible tissues via epidermal cells rather than through stomata.

### 3.2 Analysis of ROS accumulation after inoculation

**Figure 2:**
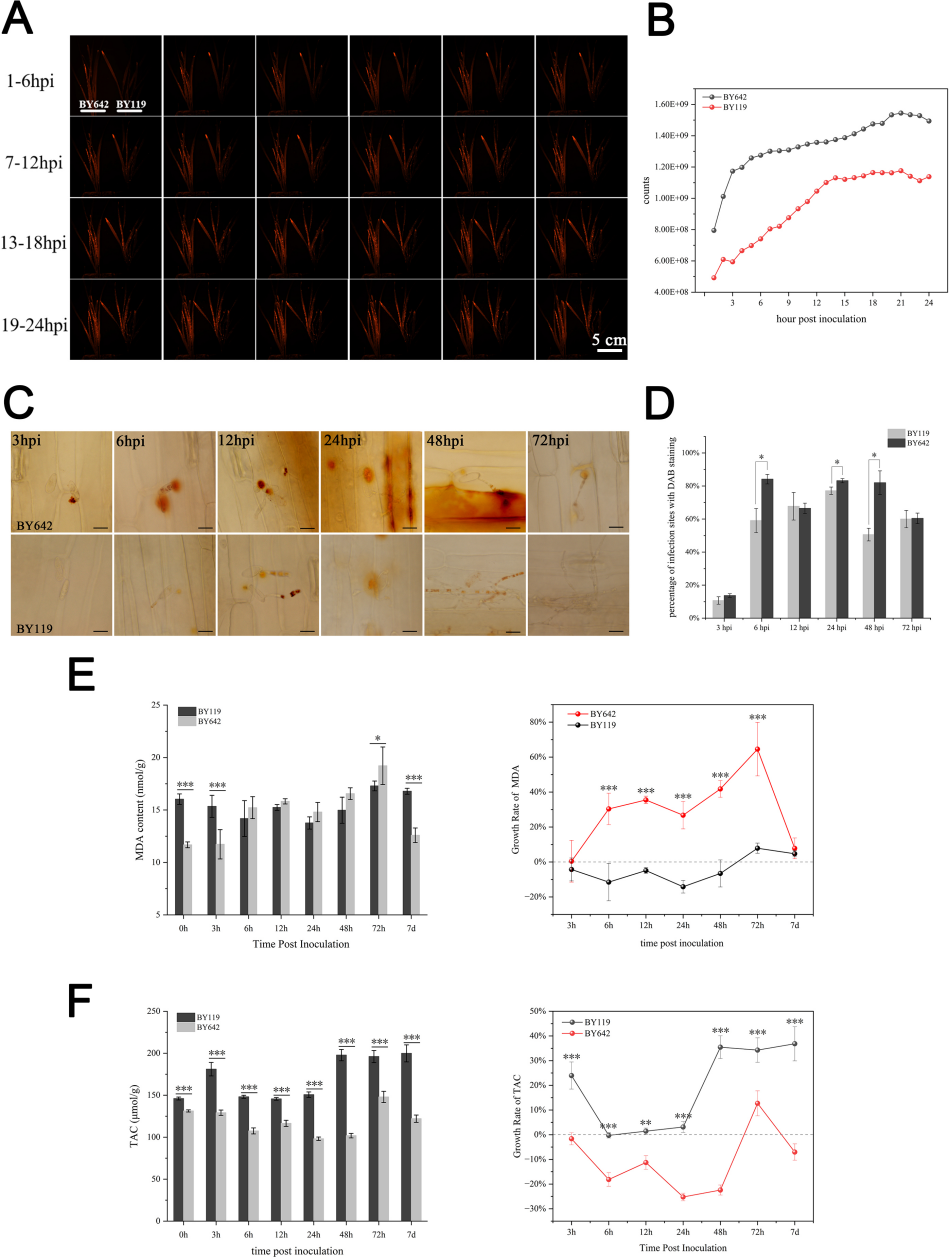
Changes in ROS production and antioxidant indicators after inoculation. **(A, C)** Histochemical staining and quantitative analysis of ROS production with 24 hours after inoculation in BY642 and BY119 by H_2_DCFDA staining. **(B)** H_2_O_2_ accumulation staining by DAB at interaction sites in BY642 and BY119 at various time points. The dark bar indicates 50μm. **(D)** Difference of H_2_O_2_ accumulation between BY642 and BY119 after inoculated. The bars represent the percentage of infection sites exhibiting H_2_O_2_ accumulation in 2 oats at various time points. Each point represents at least 100 infection sites of each of three leaf pieces. **(E)** The MDA content and growth rate. **(F)** The TAC activity and growth rate. (* represents *P* < 0.05, ** represents *P* < 0.01, *** represents *P* < 0.0001).

To investigate the role of reactive oxygen species (ROS) in the defense response of oat lines during *Bga* infection, we analyzed ROS dynamics in BY642 and BY119. Fluorescent probe analysis revealed ROS accumulation in both BY642 and BY119 after inoculation (**Figure 2A**). Quantitative analysis further showed that ROS content in BY642 was consistently higher than in BY119, especially during the first 3 hours post-inoculation, when BY642 exhibited a sharp increase in ROS levels (**Figure 2B**). To localize hydrogen peroxide (H_2_O_2_), 3,3’-diaminobenzidine (DAB) staining was performed. From 3 hpi to 12 hpi, H_2_O_2_ was primarily concentrated beneath the primary germ tubes (PGT), while at 24 hpi, it accumulated mainly below the penetration pegs (PP) in both lines (**Figure 2C**). Notably, at 24 hpi and 48 hpi, extensive H_2_O_2_ accumulation was observed in the infected and neighboring cells of BY642. In contrast, in BY119, H_2_O_2_ signals gradually weakened and disappeared by 48 hpi and 72 hpi. Quantitative assessment of H_2_O_2_ accumulation at more than 100 interaction sites revealed that BY642 exhibited significantly higher H_2_O_2_ levels than BY119 at both 6 hpi and 48 hpi (**Figure 2D**). In summary, these results indicate that the resistant line BY642 mounts a stronger and earlier ROS burst in response to *Bga* infection, potentially contributing to its enhanced defense.

### 3.3 Analysis of the antioxidant system after inoculation

To evaluate the extent of oxidative stress and membrane damage during Bga infection, we measured malondialdehyde (MDA) content and total antioxidant capacity (TAC) in BY642 and BY119. The content of MDA, an indicator of cell membrane damage under stress, varied between the two lines. Although the initial MDA levels differed, BY642 showed a significant increase from 6 hpi to 72 hpi, peaking at 72 hpi with a 64.52% rise and reaching 19.22 nmol/g (**Figure 2E**). In contrast, BY119 exhibited a decrease in MDA content from 6 hpi to 48 hpi, with the lowest point at 24 hpi (a 14.17% reduction compared to 0 h). However, its MDA levels gradually returned to near baseline by 72 hpi (7.87%) and 7 dpi (4.70%). We also assessed TAC to determine the plants’ ability to scavenge reactive oxygen species. TAC activity in BY119 was consistently higher than in BY642 across all time points (**Figure 2F**). In BY642, TAC declined within the first 48 hours, reaching its lowest value at 24 hpi (a 25.22% reduction), followed by a partial recovery (12.71% increase) at 72 hpi, and a slight decline again by 7 dpi (7.02%). Conversely, BY119 showed an early increase in TAC activity at 3 hpi (23.96%), which remained elevated by over 30% at 48 hpi, 72 hpi, and 7 dpi. In summary, these results suggest that BY642 experiences more pronounced membrane damage and a delayed antioxidant response following Bga infection, while BY119 maintains stronger basal antioxidant activity but mounts a weaker defense response, potentially contributing to its susceptibility.

### 3.4 Analysis of phytohormone after inoculation

To investigate the dynamic changes in endogenous hormone levels in BY642 and BY119 after inoculation, we quantified the concentrations of SA (salicylic acid), JA (jasmonic acid), GA (gibberellic acid), ABA (abscisic acid) and IAA (indole acetic acid) at various time points after inoculation (**Supplementary Table S2**).

**Figure 3:**
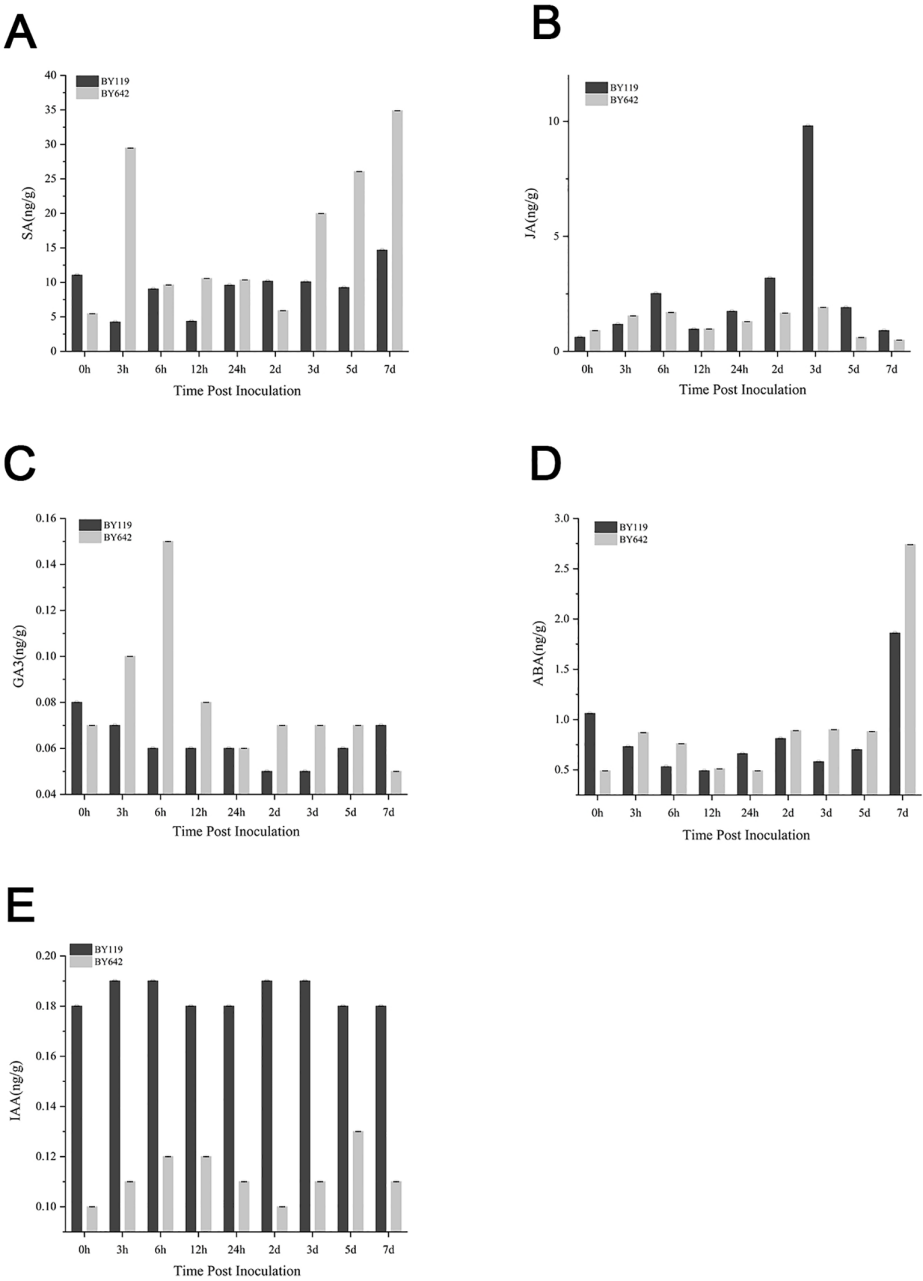
Changes in hormone levels in BY642 and BY119 after inoculation. **(A)**SA content. **(B)** JA content. **(C)** GA content. **(D)** ABA content. **(E)** IAA content.

The SA content in BY642 consistently increased after inoculation, peaking at 3 hpi with 29.46 ng/g, approximately 7 times higher than that of BY119 (**Figure 3A**). From 0 to 48 hpi, the SA levels exhibited a rise-and-fall trend, maintaining elevated levels during the later stages of infection at 3, 5, and 7 dpi. Conversely, SA levels in BY119 displayed minor fluctuations, with a marked decrease to 4.23 ng/g at 3 hpi and 4.36 ng/g at 12 hpi, both significantly lower than the pre-inoculation level (11.04 ng/g). JA levels in both BY642 and BY119 followed a rise-and-fall pattern after inoculation, and BY642 maintained consistently lower levels than BY119 from 6 hpi onward (**Figure 3B**). At 3 dpi, the JA content in BY119 sharply increased to a peak of 9.8 ng/g, far exceeding the level in BY642 (1.91 ng/g).

GA levels in both BY642 and BY119 remained relatively low post-inoculation. In BY642, GA exhibited an initial increase, peaking at 6 hpi with 0.15 ng/g, 2.5 times higher than BY119 (0.06 ng/g), before subsequently declining (**Figure 3C**). In contrast, BY119 showed an inverse trend, with GA levels initially decreasing and then gradually increasing. Similarly, ABA levels in BY642 demonstrated a rise-and-fall trend during the early stages of infection, peaking at 3 hpi with a 77.55% increase compared to the pre-inoculation level (**Figure 3D**). BY119, however, exhibited an initial decrease in ABA levels, dropping by 31.13% at 3 hpi and continuing to decline at 6 and 12 hpi. At 7 dpi, ABA levels in both varieties rose significantly. For IAA, BY119 consistently exhibited higher levels than BY642 across all time points (**Figure 3E**). Both varieties followed similar trends, with IAA levels increasing and then decreasing during the early stages of infection, followed by a secondary rise and subsequent decline during the later stages.

Overall, in the early stages of infection, BY642 exhibited a rising-then-falling trend for SA, JA, GA, ABA, and IAA levels. Among the five hormones, SA had the highest concentration. In BY119, JA and IAA levels also followed a rising-then-falling trend during the early stages of infection, whereas SA, GA, and ABA levels were lower after inoculation compared to pre-inoculation levels. These findings implied that relatively high concentrations of SA and low concentrations of JA exert pivotal functions in regulating the process of oat powdery mildew resistance response.

### 3.5 Analysis of RNA-seq, contig assembly and differentially expressed genes

To explore the dynamic transcriptional responses of resistant and susceptible oat lines during Bga infection, we performed time-course RNA sequencing on BY642 (resistant) and BY119 (susceptible) at six time points post inoculation. A total of 36 sequencing libraries (2 varieties × 6 time points × 3 replicates) were generated from the leaves of BY642 and BY119 at 0 hpi, 6 hpi, 12 hpi, 24 hpi, 48 hpi, and 156 hpi. The sequencing produced 226.34 Gb of clean reads, with Q30 values ranging from 90.68% to 94.25%, ensuring the data quality was sufficient for downstream analysis. Clean reads were mapped to the Avena sativa (cv. Sang) v1.0 reference genome, achieving alignment rates between 90.30% and 95.53%. In total, 163,459 expressed genes were identified across all libraries for further functional analysis.

To determine differentially expressed genes (DEGs), comparisons were conducted based on three biological replicates per time point. First, DEGs were identified between BY642 and BY119 at each corresponding time point, resulting in six pairwise comparisons: R0 *vs*. S0, R6 *vs*. S6, R12 *vs*. S12, R24 *vs*. S24, R48 *vs*. S48, and R156 *vs*. S156. The greatest number of DEGs was observed at 6 hpi (18,304), while the fewest were detected at 24 hpi (7,077) (**Supplementary Figure S1A**). Additionally, DEGs were analyzed within each variety by comparing post-inoculation time points to the corresponding 0 hpi samples. In BY642, the peak of transcriptional change occurred at 24 hpi, with 5,385 up-regulated and 8,656 down-regulated genes (**Supplementary Figure S1B**). In contrast, BY119 exhibited the largest number of DEGs at 48 hpi, with 7,933 genes up-regulated and 8,507 down-regulated (**Supplementary Figure S1C**). In summary, RNA-seq analysis revealed that BY642 and BY119 exhibit distinct temporal patterns of transcriptional activation in response to Bga infection, with BY642 responding more robustly at earlier time points, while BY119 shows delayed but widespread transcriptional changes.

### 3.6 Analysis of gene ontology and Kyoto Encyclopedia of genes and genomes

To eliminate the intrinsic differences between the two varieties and identify pathways with expression levels specifically increased or decreased at each time point post inoculation, we utilized Venn diagrams to analyze DEGs at each time point comparing to 0 hpi. Subsequently, GO and KEGG enrichment analyses were performed on these DEGs. The results revealed that the highest number of downregulated DEGs was observed at 6 hpi, totaling 4,417, while the largest number of upregulated DEGs was observed at 24 hpi, with 5,428 genes.

**Figure 4:**
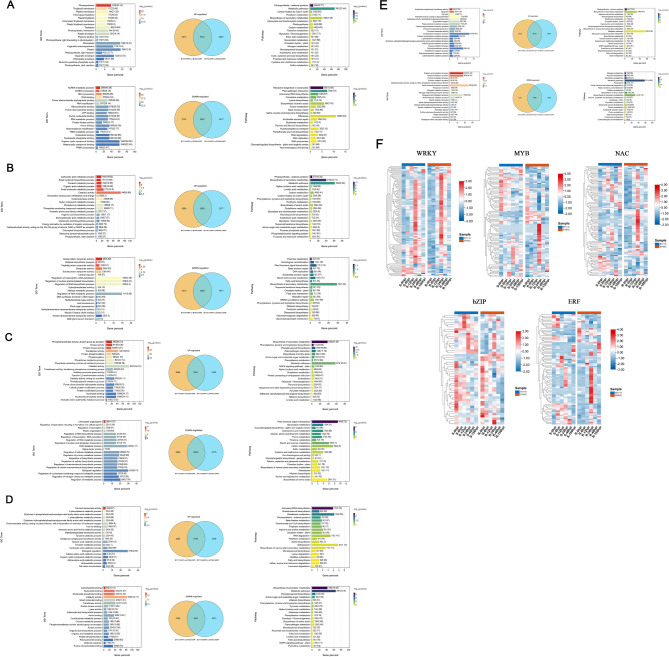
Transcriptomic analysis of enrichment pathways and gene expression dynamics of BY642 and BY119. **(A)** Gene ontology analysis and KEGG pathway of DEGs of BY642 and BY119 at 6 hpi. **(B)** Gene ontology analysis and KEGG pathway of DEGs of BY642 and BY119 at 12 hpi. **(C)** Gene ontology analysis and KEGG pathway of DEGs of BY642 and BY119 at 24 hpi. **(D)** Gene ontology analysis and KEGG pathway of DEGs of BY642 and BY119 at 48 hpi. **(E)** Gene ontology analysis and KEGG pathway of DEGs of BY642 and BY119 at 156 hpi. **(F)** Heatmaps of DEGs related to transcription factors associated with plant stress.

The GO enrichment analysis showed that at 6 hpi, 2,891 specifically upregulated DEGs were mainly enriched in Photosynthesis, Thylakoid membrane, and Plastid membrane, while 4,417 downregulated genes were predominantly associated with ncRNA- and RNA-related pathways (**Figure 4A**). At 12 hpi, 2,377 upregulated DEGs were significantly enriched in carboxylic acid metabolic process, small molecule biosynthetic process, oxoacid metabolic process, and organic acid metabolic process, with a notable increase in catalytic activity. In contrast, 1,817 downregulated DEGs were enriched in pathways related to cation, symporter, and proton processes (**Figure 4B**). At 24 hpi, 5,428 upregulated DEGs were associated with phosphotransferase activity, kinase activity, and protein kinase activity, with catalytic activity remaining significantly upregulated. Meanwhile, pathways related to chloroplast organization and sporulation regulation were downregulated, suggesting that BY642 might regulate transcription to suppress the growth of pathogen spores (**Figure 4C**). At 48 hpi, 2,796 upregulated DEGs were enriched in cis-trans isomerase activity, as well as hydrolytic metabolism of L-phenylalanine and its precursors erythrose 4-phosphate/phosphoenolpyruvate. Additionally, 2,072 downregulated DEGs were associated with carbohydrate binding and nucleotide binding. Notably, catalytic activity, kinase activity, and defense response were significantly downregulated at this time point, indicating a weakening of the disease resistance response (**Figure 4D**). At 156 hpi, 2,610 upregulated DEGs were enriched in pathways such as xyloglucan metabolism, apoplast, and cell wall, while 2,502 downregulated DEGs were associated with organic acid metabolism, oxoacid metabolism, and pullulan metabolic processes (**Figure 4E**).

The KEGG enrichment analysis demonstrated that pathways related to photosynthesis, metabolism, and secondary metabolites were significantly upregulated across multiple time points. Additionally, at 24 hpi, aromatic amino acid biosynthesis was significantly enhanced, with plant-pathogen interaction and MAPK signaling pathway also prominently upregulated (**Figure 4C**). At 48 hpi, pathways associated with aminoacyl-tRNA biosynthesis, brassinosteroid biosynthesis, and glutathione metabolism were significantly enriched (**Figure 4D**). At 156 hpi, arginine and proline metabolism were notably upregulated (**Figure 4E**). Among the downregulated DEGs, at 6 hpi, pathways were primarily enriched in ribosome biogenesis in eukaryotes and plant-pathogen interaction (**Figure 4A**). At 12 hpi, thiamine metabolism, homologous recombination, and plant hormone signal transduction were significantly downregulated (**Figure 4B**). At 24 hpi, pathways related to glycerolipid and glycosphingolipid metabolism showed downregulation (**Figure 4C**). At 48 hpi, DEGs were enriched in metabolism and secondary metabolite biosynthesis pathways (**Figure 4D**). While nitrogen metabolism, pyruvate metabolism, and glutathione metabolism were downregulated at 156 hpi (**Figure 4E**).

Furthermore, by comparing two KEGG pathways directly related to plant disease resistance, we found that at 24 hpi, most genes in the plant-pathogen interaction and MAPK signaling pathways (**Supplementary Figure S2**) were upregulated in BY642. The number of upregulated genes was also the highest at this time point compared to others. Upon analyzing the transcription factors associated with plant stress, it was observed that the expression levels of WRKY, MYB, NAC, bZIP, and ERF in BY642 were significantly upregulated at 24 hpi (**Figure 4F**).

### 3.7 WGCNA analysis and qPCR verification of hub-genes

**Figure 5:**
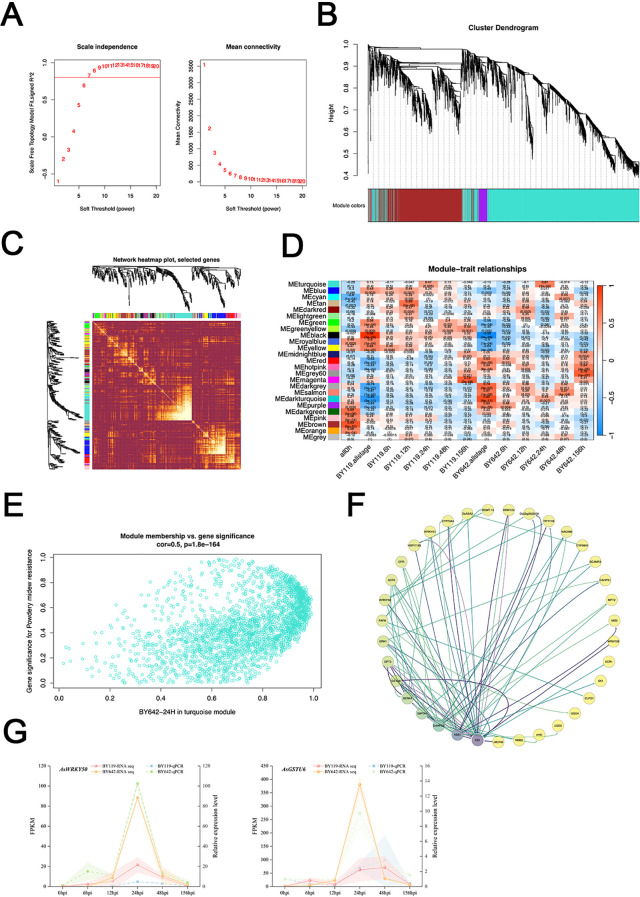
WGCNA of differentially expressed genes and qPCR Verification of Hub-genes. **(A)** Analyses of network topology for various soft-thresholding powers. **(B)** Clustering dendrogram of genes. **(C)** Visualizing the gene network using a heatmap plot, the heatmap depicts the TOM among all genes in the analysis. **(D)** Module-sample associations. **(E)** Module Membership turquoise modules. **(F)** Visualization of the network connections among the connected genes in turquoise modules generated by the Cytoscape. **(G)**Relative expression level of *AsWRKY50* and *AsGSTU6*.

To pinpoint key regulators involved in disease resistance at 24 hpi in BY642, we performed Weighted Gene Co-expression Network Analysis (WGCNA) to identify resistance-associated hub genes. Based on whole-transcriptome expression data, a scale-free network was constructed following WGCNA principles. Highly interactive gene modules were identified using Pearson correlation (r ≥ 0.85), and an optimal soft-threshold power (β = 7) was selected to ensure scale-free topology (scale-free fit index R² = 0.92), as indicated by plateaued mean connectivity (**Figure 5A**). A topological overlap matrix (TOM) was subsequently generated (**Figure 5C**), and hierarchical clustering (**Figure 5B**) divided genes into 25 co-expression modules (**Figure 5D**). Module-trait correlation analysis revealed that the turquoise module exhibited the highest positive correlation with resistance at 24 hpi (r = 0.61, P = 1×10_-4_). Within this module, 100 hub genes were identified using a combined Module Membership (MM > 0.9) and Gene Significance (GS > 0.8) filter (**Figure 5E**), indicating strong module connectivity and phenotypic relevance. Network visualization of the top 35 hub genes, ranked by GS value, highlighted candidate genes with strong potential involvement in disease resistance pathways. Nodes were colored based on GS intensity, and edges reflected TOM-based gene connectivity (**Figure 5F**). Among these, we identified hub genes *AsCS1*, *AsG6PDH*, *AsSERK4*, *AsGSTU6*, *AsERH1*, *AsASa2*, and *AsCYP98A1*, as well as transcription factors *AsWRKY24*, *AsWRKY50*, and *AsWRKY51*, which are closely related to plant responses to biotic and abiotic stress as candidate genes (**Figure 5F**). These genes are known to play important roles in plant responses to both biotic and abiotic stress, making them strong candidates for further functional validation. To validate the WGCNA results, we performed RT-qPCR analysis on selected hub genes. The expression patterns of *AsWRKY50* and AsGSTU6 were highly consistent with transcriptome data, both showing a sharp increase in expression at 24 hpi. This simultaneous upregulation suggests that *AsWRKY50* and *AsGSTU6* may be co-expressed and potentially functionally linked in mediating the resistance response (**Figure 5G**). Overall, WGCNA revealed a set of resistance-associated hub genes in BY642, with *AsWRKY50* and *AsGSTU6* emerging as promising candidate genes for powdery mildew resistance at the critical 24 hpi time point.

## 4 Discussion

Upon pathogen invasion, plants rapidly produce ROS (reactive oxygen species), leading to an oxidative burst that directly inhibits pathogen proliferation through oxidative damage (Wojtaszek, 1997). This immediate response is crucial for halting the spread of the pathogen. Beyond their direct antimicrobial effects, ROS serve as pivotal signaling molecules that activate downstream defense pathways (D’Autréaux and Toledano, 2007), including the hypersensitive response (HR) (Zurbriggen et al., 2010) and systemic acquired resistance (SAR) (Li et al., 2023a). The redox regulation of signaling components is essential for the amplification of defense signals and the establishment of long-lasting immunity. In our study, powdery mildew resistant variety “BY642” demonstrated a more robust and rapid accumulation of ROS compared to the susceptible variety “BY119”, particularly during the early stages of infection. This suggested that BY642 activates a stronger oxidative burst, an essential component of the hypersensitive response, which limits pathogen proliferation. The specific localization of H_2_O_2_ beneath PGT and PP in BY642 further highlighted its capacity to mount a targeted defense. In contrast, the gradual dissipation of ROS in BY119 after 48 hours post inoculation indicates a weaker and less sustained oxidative defense, which may contribute to its susceptibility.

ROS act as both antimicrobial agents and essential signals in plant innate immune responses, the whole process is accompanied by a tight regulation of ROS levels to balance effective defense and prevent cellular damage (Lee et al., 2020). MDA (malondialdehyde) content is commonly used as an indicator for the degree of oxidative damage to cells (Lykkesfeldt, 2007). During organ senescence or under stress conditions, plants often undergo membrane lipid peroxidation, and MDA is the terminal decomposition product of this process (Liu et al., 2019). Studies showed that ROS production leads to increased MDA levels in various plant species (Su et al., 2019; Hasanuzzaman et al., 2020). The dynamic changes in MDA content in BY642 suggested a transient imbalance in oxidative homeostasis following inoculation. The sharp initial increase in MDA levels from 6 hpi (hours post inoculation) to 72 hpi in BY642, followed by a return to baseline at 7 dpi (days post inoculation), indicated that BY642 might experience greater oxidative stress and activate a delayed recovery mechanism to restore membrane stability. It can be inferred that BY642 produces more ROS after inoculation conversely, while its overall content is not significantly higher than that of BY119, suggesting that BY642 has a better balancing mechanism to simultaneously address disease resistance and prevent self-damage. TAC (total antioxidant capacity) is a comprehensive measure of a plant’s ability to neutralize ROS which reflects the balance between ROS production and scavenging, indicating the plant’s capacity to manage oxidative stress during immune responses (Corpas et al., 2024). A robust antioxidant system is essential for maintaining cellular redox homeostasis and ensuring proper signaling during stress conditions. It always including superoxide dismutase (SOD), Peroxidase (POD), ascorbate peroxidase (APX) and catalase (CAT) (Shah and Nahakpam, 2012), as well as low molecular weight antioxidants; the ascorbate-glutathione cycle, for instance, plays a significant role in detoxifying H_2_O_2_, thereby protecting cells from oxidative damage (Kunert and Foyer, 2023). The decreased antioxidant activity in BY642 from 6 hpi to 48 hpi indicated a temporary suppression of its ability to neutralize ROS. This reduction likely contributed to the accumulation of ROS and MDA during this period. In contrast, BY119 maintained or even enhanced its antioxidant activity post-inoculation, thereby scavenging cellular ROS that might had been used to fight the invasion of powdery mildew, resulting in the smooth colonization of the fungal mycelium. These results suggested that while BY642 exhibited a rapid and pronounced oxidative response to inoculation, its capacity to regulate ROS and prevent oxidative damage was much more efficient and powerful than BY119.

SA (salicylic acid), a key hormone associated with resistance and defense against biotrophic pathogens, enhances the expression of pathogenesis-related (PR) genes, leading to ROS accumulation, calcium influx, and callose deposition. It is also crucial for establishing SAR (Alazem and Lin, 2015). Moreover, a recent study showed that H_2_O_2_ generated through NADPH oxidase activity was identified as the mobile SAR signal (Cao et al., 2024). In our study, the early SA peak at 3 hpi, followed by sustained elevation in later stages, indicated rapid activation of SA-mediated defense pathways in BY642. This observation was consistent with earlier findings on H_2_O_2_ dynamics. This contrasted sharply with BY119, in which SA levels exhibited only minor fluctuations and were lower than pre-inoculation levels during the critical early stages of infection, indicating a weaker SA-mediated defense response. JA (jasmonic acid), typically linked to necrotrophic pathogen resistance (Völz et al., 2023), exhibited distinct patterns between the two cultivars. BY642 maintained lower JA levels at almost all post-inoculation time compared to BY119, particularly at 3 dpi, which is the JA peak point in BY119. This suggested that JA-mediated defense pathways may play a less prominent role in BY642’s resistance to powdery mildew, aligning with the known antagonistic interaction between SA and JA signaling (Kazan and Manners, 2013). The sharp increase in JA levels in BY119 may reflect a stress response rather than an effective defense mechanism. Dynamic changes in GA (gibberellic acid) and ABA (abscisic acid) levels further underscored the role of hormonal crosstalk in regulating infection responses. In BY642, both GA and ABA levels followed a rising-then-falling pattern during the early stages of infection, suggesting their involvement in modulating early defense responses. However, in BY119, the initial decrease in GA and ABA levels may reflect a delay or suppression of their roles in coordinating stress responses. In summary, the hormonal dynamics in BY642 demonstrate a well-coordinated defense strategy dominated by SA signaling, with appropriate modulation of other hormonal pathways.

The RNA-seq data revealed temporal and quantitative differences in gene expression after powdery mildew inoculation between the two varieties. At 6 hpi, upregulated DEGs (differentially expressed genes) in photosynthesis and membrane processes, along with downregulation of ncRNA and RNA-related pathways, suggest an early shift toward energy production and cellular integrity. By 12 hpi, increased organic acid metabolism and catalytic activity indicate enhanced metabolic reconfiguration for defense compound biosynthesis. The marked increase in upregulated DEGs associated with kinase activity, phosphotransferase activity, and plant-pathogen interaction pathways at 24 hpi reveals the activation of robust signaling networks in BY642. The simultaneous downregulation of pathways related to chloroplast organization and sporulation regulation suggests that BY642 may actively repress pathogen growth while enhancing its own immune signaling. This aligns with the observed upregulation of MAPK signaling, aromatic amino acid biosynthesis, and other secondary metabolic pathways, which are critical for producing defense-related metabolites. At 48 hpi, the enrichment of down-regulated DEGs in pathways like catalytic and kinase activities, as well as defense responses, indicated a potential decline in immune activity. This shift may prioritize energy conservation and activate cellular repair mechanisms post-defense. By 156 hpi, the upregulation of pathways related to cell wall modification, such as xyloglucan metabolism and apoplast-associated processes, suggested a strengthening of structural defenses to limit further pathogen spread.

Several hub genes play a key role at 24 hpi. *AsGSTU6* encodes a glutathione S-transferase(GST), an enzyme that suppresses ROS-scavenging genes to maintain intracellular ROS homeostasis, thereby enhancing cadmium stress tolerance in rice (Jing et al., 2021). Studies showed that GSTs form a versatile superfamily of enzymes that play essential roles in secondary metabolism and in providing resistance to environmental stress (Cui et al., 2021). The most sensitive targets for H_2_O_2_-dependent oxidation are thiol peroxidases of the peroxiredoxin (PRX) or glutathione peroxidase (GPX) families (Dietz and Vogelsang, 2022). GPX functions as antioxidant enzyme, utilizing glutathione to reduce hydroperoxides (Crawford et al., 2012). *AsWRKY50* also emerged as a key regulator. WRKY TFs are well-documented mediators of plant hormone signaling and are critical in modulating responses to biotic and abiotic stresses (Jiang et al., 2017; Wang et al., 2021; Khoso et al., 2022; Yan et al., 2022). *AsWRKY50* may coordinate the activation of downstream defense-related genes in BY642, enhancing its ability to resist pathogen attack. in Arabidopsis, MPK3/MPK6 regulate the expression of downstream defense genes through phosphorylation of WRKY transcription factors (Wang et al., 2023). A similar function may be attributed to *AsWRKY50* in BY642: upon phosphorylation by upstream kinases, it translocates to the nucleus, where it directly binds to the W-box elements in the promoters of PR protein genes, activating the expression of pathogenesis-related proteins. Based on their strong expression induction upon pathogen invasion and consistent transcriptome and RT-qPCR validation results, *AsGSTU6* and *AsWRKY50* represent promising candidate genes for powdery mildew resistance breeding in oat. Given their specific and significant upregulation in the resistant line BY642 at 24 hpi, these genes could serve as molecular markers for early selection of resistant genotypes if polymorphic sites (e.g., SNPs or InDels) are identified within or near their coding or regulatory regions. Furthermore, considering the established roles of GSTs and WRKY transcription factors in plant defense signaling pathways, these genes also show potential as transgenic targets to enhance resistance in susceptible cultivars. Future functional studies, such as gene overexpression or knockout assays, would further clarify their roles and facilitate their application in marker-assisted selection (MAS) and genetic transformation strategies aimed at improving powdery mildew resistance in oat.

Besides *AsGSTU6* and *AsWRKY50*, the following genes may also play important roles. *AsSERK4*, a member of the leucine-rich repeat receptor-like protein kinase (LRR-RLK) family, contributes to plant immunity through PTI activation, MAPK signaling, and ROS accumulation (Shiu and Bleecker, 2001). Previous studies demonstrated that *AtSERK4* regulates cell death (Gou et al., 2012) and innate immunity (Li, 2010), supporting *AsSERK4*’s potential role in BY642’s resistance. Furthermore, *AsERH1* is associated with SA-induced powdery mildew resistance in Arabidopsis (Wang et al., 2008); *AsCYP98A1*, a cytochrome P450 enzyme, is involved in the phenylpropanoid pathway and response to SA derivatives (Basson and Dubery, 2007; Karamat et al., 2012); AsG6PDH plays a crucial role in NADPH production and redox homeostasis via the pentose phosphate pathway (Moraes et al., 2023). These aforementioned genes serve as potential candidates for powdery mildew resistance in BY642, functioning crucially either directly or indirectly during the plant’s defense response.

**Figure 6:**
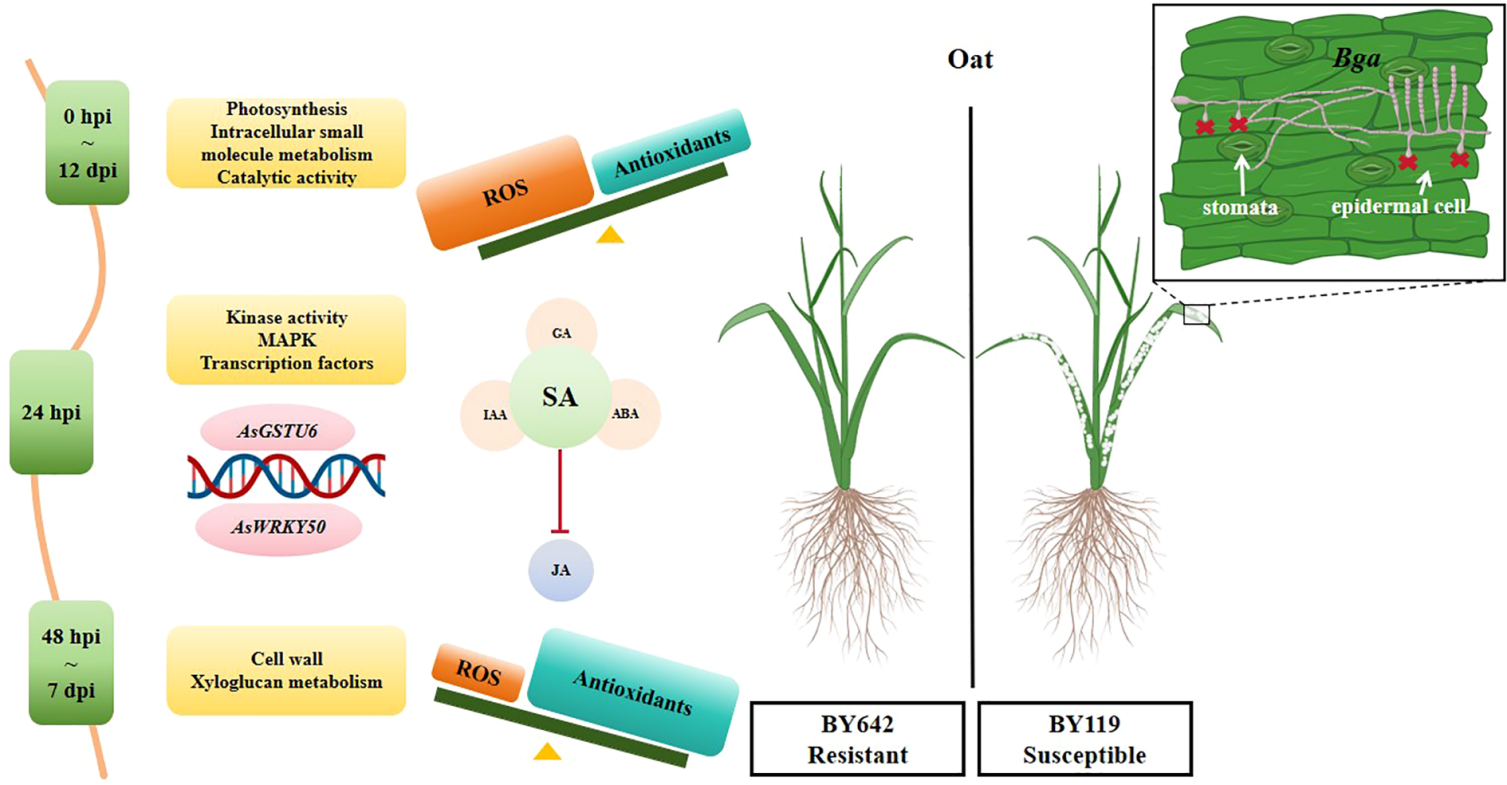
Graphical summary of physiological and molecular mechanisms underlying powdery mildew resistance in BY642.

In conclusion, this study provides insights into the physiological responses and molecular mechanisms underlying powdery mildew resistance in BY642 (**Figure 6**). Upon inoculation, BY642 rapidly accumulated ROS and efficiently regulated antioxidants to maintain redox balance, with an SA-dominated signaling pathway and suppressed JA involvement coordinating its immune response. The molecular mechanisms encompass early activation of photosynthesis, metabolic reprogramming, and immune pathways, including the MAPK pathway and WRKY transcription factors, along with the expression of key hub genes such as *AsGSTU6* and *AsWRKY50*.

## Data Availability

The raw sequence data reported in this paper have been deposited into CNGB Sequence Archive (CNSA) (Guo et al., 2020) of China National GeneBank DataBase (CNGBdb) (Chen et al., 2020) with accession number CNP0006917.
