# Supplementary material for: Physiological and molecular profiling unveils oat (Avena sativa L.) defense mechanisms against powdery mildew
Source: Front Plant Sci. 2025 May 8;16:1580472. doi: 10.3389/fpls.2025.1580472 (PMC12095171; doi:10.3389/fpls.2025.1580472)
Supplement: Supplementary file 1 [file DataSheet1.docx]

Supplementary Material


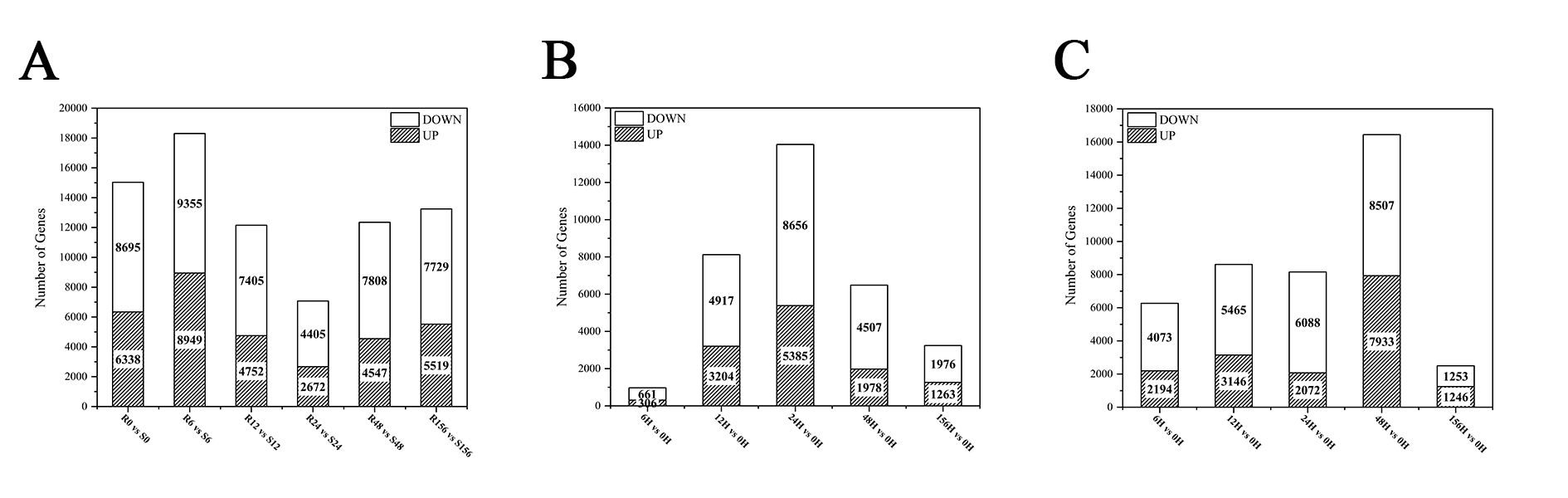


Figure S1. Comparative analysis of DEGs in BY642 (R) and BY119 (S) after inoculation at different time points. (A) Number of DEGs between BY642 (R) and BY119 (S) at different time points. (B) DEGs after inoculation in BY642 compared to 0h. (C) DEGs after inoculation in BY119 compared to 0h.


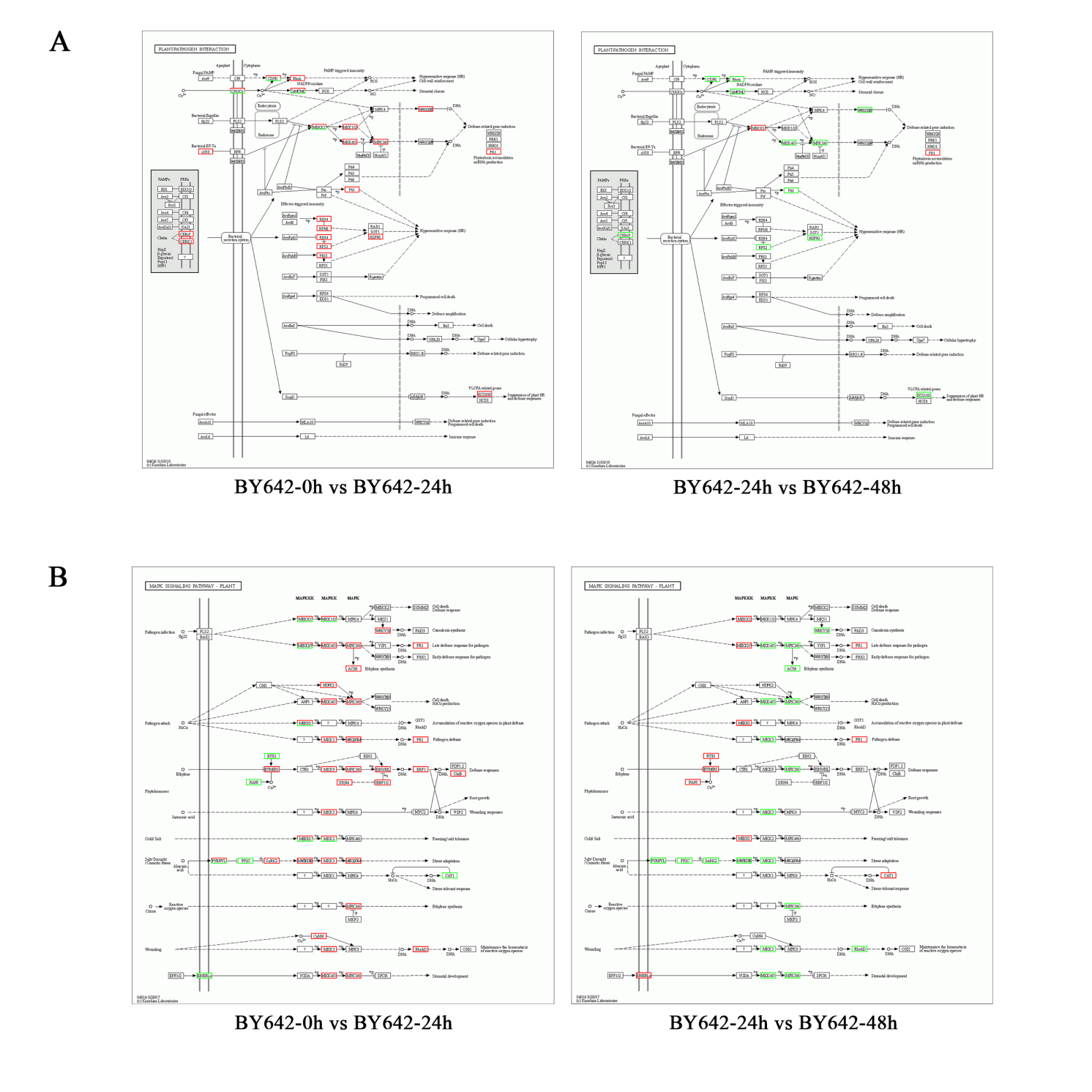


Figure S2. Plant-pathogen interaction (A) and MAPK signaling pathway (B) in BY642. The red box represents up-regulated genes, and the green represents the down-regulated genes.

Table S1. The Primers used in the study of RT-qPCR analysis

| Gene ID | Gene | F | R |
| --- | --- | --- | --- |
| AVESA.00010b.r1.4CG1326820 | *AsCS1* | TGGGCTTGGCACTCCTGT | GAGCGATTGGTTCGTGTTCT |
| AVESA.00010b.r1.1DG0138170 | *AsOsASA2* | ACAGTGGCGGGTTAGGAG | TCGTGTTGTGGCTTGGAG |
| AVESA.00010b.r1.7CG0698170 | *AsSERK4* | ACAAGATTACTGGTGGCA | AGGAATAGACCCATTTAGAC |
| AVESA.00010b.r1.2AG0227370 | *AsG6PDH* | TCCGTGAACCCAATAAAG | AGTCATCTGGCACCGTAG |
| AVESA.00010b.r1.1AG0071420 | *AsWRKY24* | CTCCTTTCAGGCGGTCAA | GCGACGCTCTTGCTTACTTG |
| AVESA.00010b.r1.1AG0039340 | *AsWRKY51* | CGGAAGGGTGCGACGTGAA | GTGGCGTAGTAGACCGTGCTG |
| AVESA.00010b.r1.1AG0060910 | *AsGSTU6* | TGCTCCTCCAGTCCAACCC | CGATGAAAGCAGCCCAGAA |
| AVESA.00010b.r1.1AG0071920 | *AsERH1* | TATCATTGCCGTGAGGGTT | ATCAAGTCGCCACATCCA |
| AVESA.00010b.r1.1AG0012840 | *AsCYP98A1* | ATCATCTCCGTCTGGTTCG | GAGCTGCTGGTCTTTCTCC |
| AVESA.00010b.r1.1AG0039330 | *AsWRKY50* | GGAAGGGTGCAACGTGAAGA | CGTCCTGGCTGGCGTAGTAGA |

Table S2. Plant hormone contents of BY642 and BY119 at different time points

| **Sample** | **Sample Weight (g)** | **IAA (ng/g)** | **ABA (ng/g)** | **JA (ng/g)** | **SA (ng/g)** | **GA3 (ng/g)** |
| --- | --- | --- | --- | --- | --- | --- |
| BY119-0 hpi | 1.061 | 0.19 | 0.81 | 3.18 | 10.15 | 0.05 |
| BY642-0 hpi | 1.075 | 0.10 | 0.89 | 1.66 | 5.90 | 0.07 |
| BY119-3 hpi | 1.021 | 0.19 | 0.73 | 1.17 | 4.23 | 0.07 |
| BY642-3 hpi | 1.102 | 0.11 | 0.87 | 1.54 | 29.46 | 0.10 |
| BY119-6 hpi | 1.070 | 0.19 | 0.53 | 2.51 | 9.03 | 0.06 |
| BY642-6 hpi | 1.025 | 0.12 | 0.76 | 1.69 | 9.62 | 0.15 |
| BY119-12 hpi | 1.066 | 0.18 | 0.49 | 0.96 | 4.36 | 0.06 |
| BY642-12 hpi | 1.054 | 0.12 | 0.51 | 0.97 | 10.56 | 0.08 |
| BY119-24 hpi | 1.061 | 0.18 | 0.66 | 1.74 | 9.58 | 0.06 |
| BY642-24 hpi | 1.078 | 0.11 | 0.49 | 1.29 | 10.36 | 0.06 |
| BY119-2 dpi | 1.055 | 0.18 | 1.06 | 0.61 | 11.04 | 0.08 |
| BY642-2 dpi | 1.029 | 0.10 | 0.49 | 0.90 | 5.45 | 0.07 |
| BY119-3 dpi | 1.098 | 0.19 | 0.58 | 9.80 | 10.07 | 0.05 |
| BY642-3 dpi | 1.096 | 0.11 | 0.90 | 1.91 | 19.98 | 0.07 |
| BY119-5 dpi | 1.046 | 0.18 | 0.70 | 1.90 | 9.24 | 0.06 |
| BY642-5 dpi | 1.049 | 0.13 | 0.88 | 0.60 | 26.06 | 0.07 |
| BY119-7 dpi | 1.040 | 0.18 | 1.86 | 0.90 | 14.66 | 0.07 |
| BY642-7 dpi | 1.094 | 0.11 | 2.74 | 0.49 | 34.88 | 0.05 |
